# Preparation of Micro-Nano Material Composed of Oyster Shell/Fe_3_O_4_ Nanoparticles/Humic Acid and Its Application in Selective Removal of Hg(II)

**DOI:** 10.3390/nano9070953

**Published:** 2019-06-30

**Authors:** Chuxian He, Junhao Qu, Zihua Yu, Daihuan Chen, Tiantian Su, Lei He, Zike Zhao, Chunxia Zhou, Pengzhi Hong, Yong Li, Shengli Sun, Chengyong Li

**Affiliations:** 1 School of Chemistry and Environment, Guangdong Ocean University, Zhanjiang 524088, China; 2 Shenzhen Institute of Guangdong Ocean University, Shenzhen 518108, China; 3 College of Food Science and Technology, Guangdong Ocean University, Zhanjiang 524088, China; 4 Guangdong Modern Agricultural Science and Technology Innovation Center, Zhanjiang 524088, China

**Keywords:** mercury adsorption, humic acid, Fe_3_O_4_ nanoparticles, oyster shell

## Abstract

Micro-nano composite material was prepared to adsorb Hg(II) ions via the co-precipitation method. Oyster shell (OS), Fe_3_O_4_ nanoparticles, and humic acid (HA) were used as the raw materials. The adhesion of nanoparticles to OS displayed by scanning electron microscopy (SEM), the appearance of the (311) plane of standard Fe_3_O_4_ derived from X-ray diffraction (XRD), and the transformation of pore sizes to 50 nm and 20 μm by mercury intrusion porosimetry (MIP) jointly revealed the successful grafting of HA-functionalized Fe_3_O_4_ onto the oyster shell surface. The vibrating sample magnetometer (VSM) results showed superparamagnetic properties of the novel adsorbent. The adsorption mechanism was investigated based on X-ray photoelectron spectroscopy (XPS) techniques, which showed the process of physicochemical adsorption while mercury was adsorbed as Hg(II). The effects of pH (3–7), initial solution concentration (2.5–30 mg·L^−1^), and contact time (0–5 h) on the adsorption of Hg(II) ions were studied in detail. The experimental data were well fitted to the Langmuir isotherm equation (R^2^ = 0.991) and were shown to follow a pseudo-second-order reaction model (R^2^ = 0.998). The maximum adsorption capacity of Hg(II) was shown to be 141.57 mg·g^−1^. In addition, this new adsorbent exhibited excellent selectivity.

## 1. Introduction

Mercury pollution is considered a pervasive problem in atmospheric, aquatic, and edaphic systems. Inorganic mercury derives from a wide range of anthropogenic and natural sources, including fuel combustion, chlor-alkali plants, battery industries, coal mining, and volcanic eruption, resulting in pulmonary disorder [1] and immune system dysfunction [2]. Moreover, the conversion of methylmercury by inorganic mercury leads to a series of physical problems. For example, it can accumulate in humans through seafood consumption and cause cardiovascular and neurological disorders [3]. The traditional methods of heavy metal removal include ion exchange [4], chemical precipitation [5], membrane filtration [6], material adsorption [7], coagulation–flocculation [8], ion and precipitate flotation [9], and electrochemical treatment [10]. As the main branch of mercury removal, material adsorption has its unique superiorities, including advanced treatment even at low concentration levels and environmentally friendly properties.

Magnetic nanoparticles can remove metal ions quickly and separate from water easily due to their large surface area and strong magnetic properties, which have recently drawn researchers’ attention [11,12]. Fe_3_O_4_/humic acid (HA) was observed to have the superiority in removing heavy metal ions. Previous research literature reported the tight affinity of HA with Fe_3_O_4_ nanoparticles via abundant phenolic hydroxyl, carboxylic acid, and quinone functional groups [13,14,15]. The combination of HA on the surface of the metal oxide forms a polyanionic organic coating, which changes the surface properties of the particles and thus affects their adsorption capacity [16]. For most metal ions, the superimposed effect enhances the adsorption capacity compared with pristine Fe_3_O_4_ nanoparticles and HA.

As the largest oyster consumer, China has suffered from severe concerns about the hygiene of waste oyster shells [17]. The utilization of oyster shell (OS) not only helps the government tackle environmental issues but also increases its added values. Owing to its advantages such as low cost, sustainability, nontoxicity to humans, and environmental friendliness, OS is expected to be an appropriate natural precursor material [18,19]. With its porous structure, modified OS is commonly used to adsorb heavy metals and dyes [20,21,22,23]. However, its adsorption capacity is limited. Recent research showed that the composition of core carbon structures could increase special surface area, possibly leading to a significant breakthrough in the adsorption process [24].

In the present study, we fabricated a low-cost adsorbent via co-precipitation and used oyster shell as a carrier to load humic acid-modified Fe_3_O_4_ nanoparticles. The characterizations of the adsorbents were measured and the adsorption behaviors were investigated. The impact of pH, initial concentration of Hg^2+^ ions, reaction time, surface functionality, and metal ion species were also examined. Moreover, a possible mechanism of mercury adsorption by OS/Fe_3_O_4_/HA was discussed. The study first synthesized a novel material composed of oyster shell, Fe_3_O_4_ nanoparticles, and humic acid, which showed extraordinary capacity and selectivity in mercury adsorption and helped the reuse of kitchen waste. Thus, this material may provide a novel route to pre-concentrate or separate Hg(II) ions in the chlor-alkali process and play an important role in environmental remediation.

## 2. Experimental Section

### 2.1. Preparation of Oyster Shell (OS)

OS was collected from Zhanjiang’s sea market, washed carefully for several times, dried using an electric thermostatic drying oven, and then knocked into pieces as small as possible (r ≤ 0.15 cm). After the procedures of ball mill grinding (12 h) and vacuum freeze-drying (48 h), the fragments of OS were converted into porous powders and stored in a refrigerator at −22 °C in preparation for the synthesis of the composite adsorbent.

### 2.2. Synthesis of Magnetic Nanomaterials

Fe_3_O_4_ magnetic nanoparticles with specific modifiers were synthesized by the co-precipitation method as previously reported [25,26]. In brief, 6.1 g of FeCl_3_·6H_2_O and 4.2 g of FeSO_4_·7H_2_O were added to 100 mL of ultrapure water. The iron-mixed solution was heated to 90 °C while stirring constantly. Then 10 mL of ammonium hydroxide, 0.25 g of HA, and 0.25 g of shell powder were dissolved in 50 mL of ultrapure water and added to the reactive liquid. The mixture was magnetically stirred for 1 h at 90 °C and then cooled down to room temperature. The acquired magnetic micro-nano material (OS/Fe_3_O_4_/HA) was collected by a strong magnet and washed with ultrapure water three times. The material was stored in an aqueous solution.

The preparation of Fe_3_O_4_/HA and Fe_3_O_4_/OS adsorbents was the same as that for OS/Fe_3_O_4_/HA except for the amount of added material. In the process of synthesizing Fe_3_O_4_/HA, 0.5 g of HA was dissolved in 50 mL of ultrapure water and added to the reactive liquid. In the process of synthesizing Fe_3_O_4_/OS, 0.5 g of OS was mixed in 50 mL of ultrapure water and added to the reactive liquid. The concentration of the solution was measured after preparation.

### 2.3. Adsorption Experiments 

To determine the adsorption behavior of OS/Fe_3_O_4_/HA, a series of adsorption experiments were carried out on a magnetic stirrer. First, 1.5 mL of adsorbent was dispersed in 100 mL of a standard aqueous Hg(II) ion solution with a specialized concentration. The reactive solution was collected with a strong magnet placed under the reaction vessel, and then the analysis was tested by an atomic fluorescence spectrometer. In this study, the effects of pH (3–7), initial Hg(II) ion concentration (2.5–30 mg·L^−1^), and reaction time (0–5 h) were investigated to figure out the adsorption isotherms and kinetics. The experiments were repeated three times.

Shown below is the equation of the Hg(II) adsorption capacity *q_e_* (mg·g^−1^), which is used to describe the extent of the adsorption reaction.

(1) $$q_{e}=\frac{\left(C_{0}-C_{e}\right)V}{m}$$

where *C*_0_ and *C_e_* (mg·L^−1^) denote the initial and equilibrium concentrations, respectively. *V* (L) is the volume of the reactive solution and *m* (g) represents the dry weight of the adsorbent we used.

### 2.4. Experimental Analyses

The X-ray diffraction (XRD) measurements were carried out using a D8 ADVANCE powder X-ray diffractometer in the 2θ range of 10–90° to examine the composition of the material. Scanning electron microscopy (SEM) was performed using a Hitachi S-4800 to examine the morphology of adsorbents. The Brunauer–Emmett–Teller (BET) method was used to calculate the specific surface areas by QUADRASORB SI. Mercury intrusion porosimetry (MIP) was performed using a Quantachrome poremaster-33 mercury intrusion porosimeter to ensure the pore size distribution. A vibrating sample magnetometer (VSM, Quantum Design PPMS-9) was employed to investigate the magnetic properties of the magnetic material. X-ray photoelectron spectroscopy (XPS) was conducted via a Thermo scientific ESCALAB 250XI multifunctional imaging electron spectrometer to identify the interaction between the adsorbent and adsorbate.

## 3. Results and Discussion

### 3.1. Material Characterization

**Figure 1:**
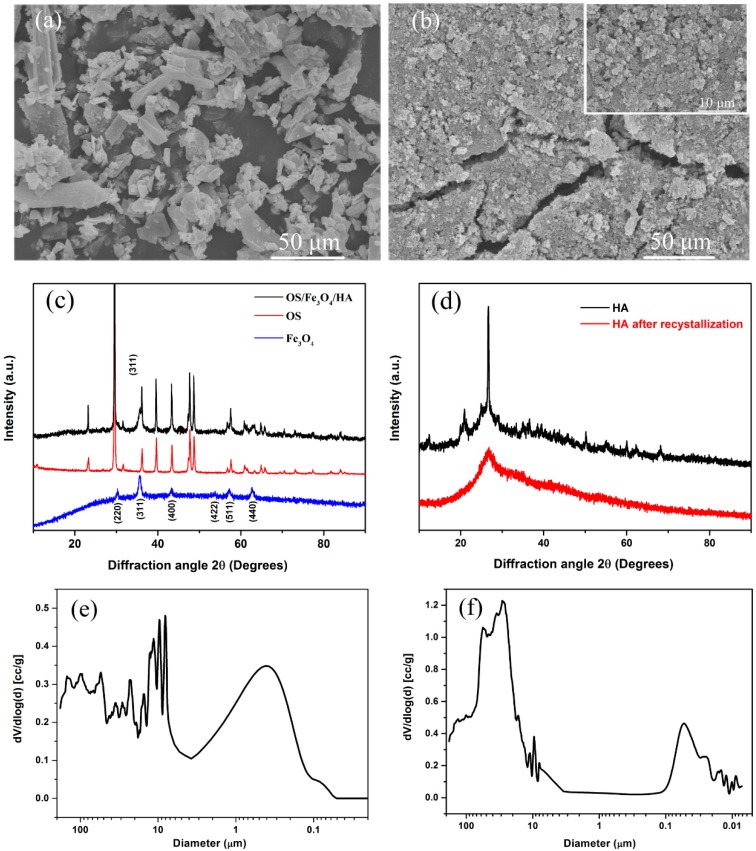
SEM images of (**a**) oyster shell (OS) and (**b**) OS/Fe_3_O_4_/humic acid (HA). X-ray diffraction (XRD) patterns of (**c**) OS/Fe_3_O_4_/HA and its constitutions of OS, Fe_3_O_4_, and (**d**) HA. Intrusion curves of (**e**) OS and (**f**) HA/Fe_3_O_4_/OS (Hg contact angle: 140°).

SEM micrographs of OS and OS/Fe_3_O_4_/HA are shown in Figure 1a,b, respectively. As the figures indicate, OS is composed of irregular sheet-layered architectures with a porous structure to load magnetic nanoparticles. In the process of synthesizing the composite adsorbent, the modified nanoparticles, which reveal a clear tendency to aggregate, tightly attach to the surface and the pore channel of OS. With the encapsulation of HA-modified Fe_3_O_4_, the quantity of active sites increases sharply.

Figure 1c shows the XRD patterns of OS/Fe_3_O_4_/HA, OS, Fe_3_O_4,_ Fe_3_O_4_/HA. The diffraction pattern of Fe_3_O_4_ exhibited six peaks at 30.2°, 35.6°, 43.3°, 53.7°, 57.3°, and 62.8°, which correspond to the (220), (311), (400), (422), (511), and (440) planes of the standard Fe_3_O_4_ [27]. In addition, the characteristic reflections of the (221), (210), and (213) planes corresponding to γ-Fe_2_O_3_ were absent, which indicates that the preparation of Fe_3_O_4_ nanoparticles had a high purity [28,29]. The diffraction pattern of OS/Fe_3_O_4_/HA not only showed similar peaks to those of OS, but it also displayed the characteristic peak of Fe_3_O_4_ nanoparticles at 35.6°. However, the peaks of HA were missed. This phenomenon may be due to the recrystallization of HA, which results in the conversion of crystal structure to an amorphous type, as the broad peak appeared in Figure 1d.

The BET and MIP methods are always used to investigate the physical properties of materials. The BET results exhibited that the specific surface areas of OS and HA/Fe_3_O_4_/OS were 7.02 m^2^·g^−1^ and 47.48 m^2^·g^−1^, respectively. The MIP method is commonly used to analyze mesoporous and macroporous materials. The intrusion curves of OS and OS/Fe_3_O_4_/HA via the MIP method exhibited different pore size distributions, as shown in Figure 1e,f. The OS curve showed two peaks at about 400 nm and 10 μm while the OS/Fe_3_O_4_/HA curve revealed two peaks at about 50 nm and 20 μm. The appearance of a smaller pore size may be attributed to the formation of nanoparticles, and the appearance of a larger pore size may be due to the effect of particle agglomeration [30].

The hysteresis loop (Figure S1) was obtained by a VSM, revealing no hysteresis, minimal remanence, and minimal coercivity, which shows its superparamagnetic characteristic. Also, the magnetization of OS/Fe_3_O_4_/HA reached 68.77 emu/g in the saturation state. The superparamagnetic behavior and strong ferromagnetism suggest that the adsorbent could be facilely separated from the aqueous solution under strong magnetism and disperse quickly the moment it disappeared [31].

### 3.2. Optimization of pH in Hg(II) Ion Adsorption

The pH can affect the extent of hydrolysis of Hg(II) ions, which will interrupt the calculation of the adsorption capacity of the adsorbent. Also, it influences the interfacial chemistry of adsorbents. Previous reports indicate that Hg(OH)_2_ replaced Hg^2+^ and was predominant in the solution in a near-neutral pH environment [32]. In addition, the calcium carbonate in the OS dissolved, thereby making the composite adsorbent unstable in a strongly acidic aqueous system [33]. Thus, the reactive pH range of 3–7 was selected to detect the course of the adsorption reaction.

**Figure 2:**
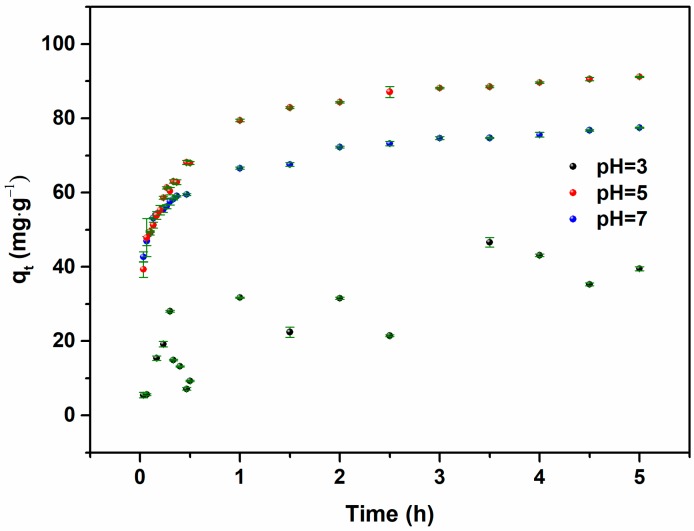
Effect of pH values on the adsorption capacity of Hg^2+^ ions via OS/Fe_3_O_4_/HA (initial Hg(II) ion concentration, 25 mg·L^−1^, contact time, 5 h).

The adsorption capacity of OS/Fe_3_O_4_/HA was investigated. As shown in Figure 2, the adsorption quantity reached the maximum value under the condition of pH = 5 when equilibration was achieved. With the increase of pH, the deprotonation of adsorption sites and the structural damage of HA affected the attraction between the adsorbent and Hg(II) ions jointly. The decrease in mercury ion adsorption at pH = 7 indicated that HA had a greater effect on mercury removal. Under a low pH condition, the excess H^+^ competed with Hg(II) ions for adsorption sites and destroyed the structure of the OS, thus lowering the adsorption capacity of Hg(II) ions. Therefore, pH = 5 was selected to carry out the following experiment.

### 3.3. Adsorption Isotherms

**Figure 3:**
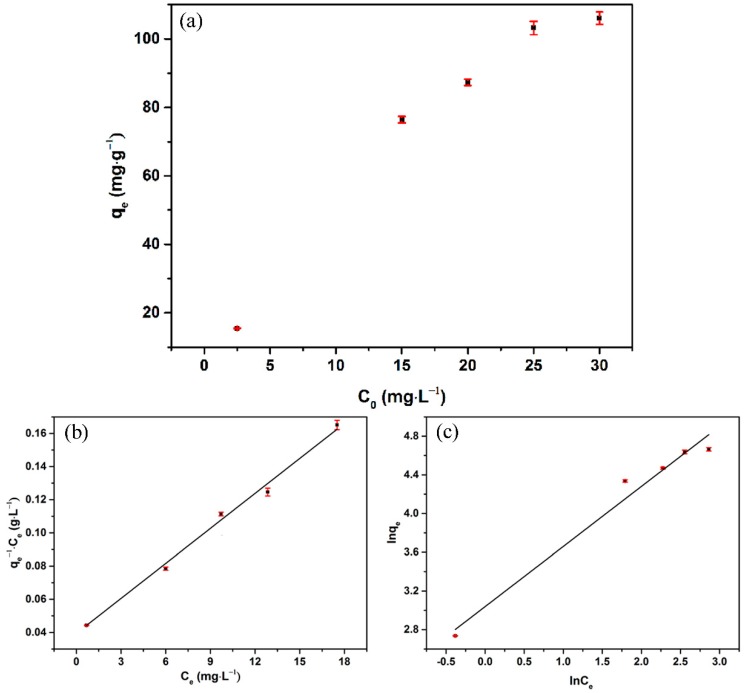
(**a**) Adsorption isotherms of HA/Fe_3_O_4_/OS, according to (**b**) the Langmuir equation and (**c**) the Freundlich equation (pH = 5; contact time, 24 h).

Figure 3a shows the impact of different initial concentrations on the adsorption efficiency. The adsorption capacity exhibited persistent enhancement with the augmentation of the initial concentration. With the increase in initial concentration, the increasing drive force promoted the adsorption reaction [34]. The Langmuir and Freundlich isotherms were used to explore the properties of OS/Fe_3_O_4_/HA. The Langmuir isotherm is based on the monolayer adsorption onto the chelating sites that are allowed to be occupied only once [35]. The equation is represented as follows:

(2) $$\frac{C_{e}}{q_{e}}=\frac{C_{e}}{q_{max}}+\frac{1}{bq_{max}}$$

where *q_e_* and *C_e_* represent the adsorption amount of Hg(II) ions (mg·g^−1^) by HA/Fe_3_O_4_/OS and Hg(II) ion concentration (mg·L^−1^) in the solution, respectively. *b* and *q_max_* are the Langmuir constant (L·mg^−1^) and the maximum saturated adsorption capacity (mg·L^−1^) that can be calculated from the linear plot of *q_e_*^−1^*C_e_* versus *C_e_*.

Different from the Langmuir isotherm, the Freundlich isotherm is always used to denote the heterogeneous systems that refer to the heterogeneous binding sites on the adsorbent surface, and can be expressed by Equation (3) [36].

(3) $$\ln q_{e}=\ln k_{f}+\frac{\ln C_{e}}{n}$$

where *k_f_* is the Freundlich adsorption constant (mg·g^−1^) decided by bond strength and *n*^−1^ represents the adsorption intensity (dimensionless). The value of these two parameters is governed by the intercept and slope of the linear plot of ln *q_e_* versus ln *C_e_*. *n* > 1 means that the adsorption process is favorable [22].

The fitting curves (Figure 3b,c) and their regression coefficient (R^2^) shown in Table 1 imply that the Langmuir model is more suitable than the Freundlich model to describe the adsorption equilibrium behavior of Hg(II) ions onto the adsorbent. Therefore, it can be assumed that the adsorption behavior occurs at single-layer adsorption.

### 3.4. Adsorption Kinetics

**Figure 4:**
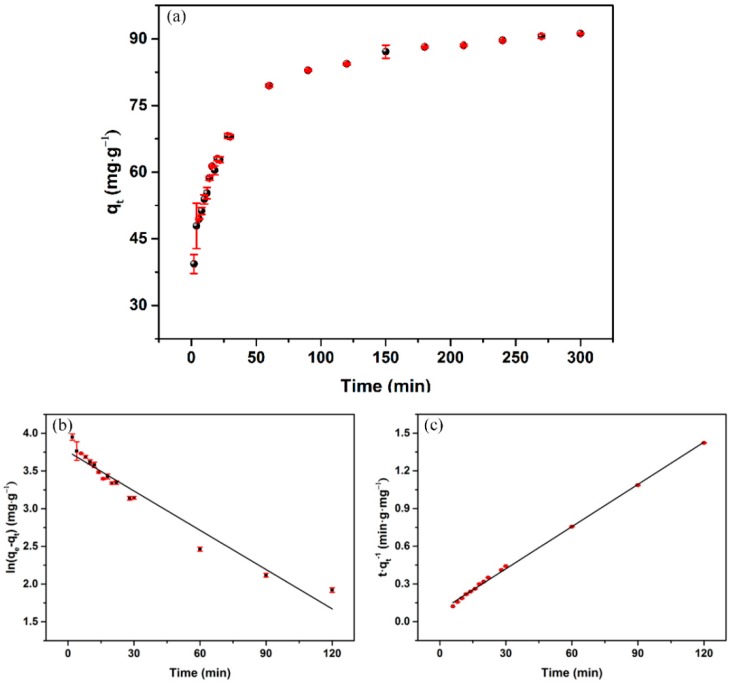
(**a**) Adsorption kinetics of HA/Fe_3_O_4_/OS, based on (**b**) the pseudo-first-order model and (**c**) the pseudo-second-order model (initial Hg(II) ion concentration, 25 mg·L^−1^, pH = 5).

The kinetics curve of Hg(II) ion adsorption on OS/Fe_3_O_4_/HA was obtained by contacting with 25 mg·L^−1^ of Hg(II) ions at pH = 5. Figure 4a reveals that the adsorption reaction occurred rapidly within 30 min and the increase in *q_t_* was not significant after 60 min. This may be due to the fast diffusion from Hg(II) ions in the solution to active sites of the adsorbent. The adsorption quantity reached a climax (91.2 mg·g^−1^) at equilibrium when the binding sites were totally occupied. The adsorption kinetics curve was further analyzed with the pseudo-first-order model and the pseudo-second-order model, using Equations (4) and (5) [37].

(4) $$\ln\left(q_{e}-q_{t}\right)=\ln q_{e}-k_{1}t$$

(5) $$\frac{t}{q_{t}}=\frac{1}{k_{2}q_{e}^{2}}+\frac{t}{q_{e}}$$

where *q_t_* and *q_e_* represent the adsorption capacity of Hg(II) ions on the fabricated OS at an arbitrary time and at equilibrium, respectively. *k*_1_ and *k*_2_ represent the pseudo-first-order model rate constant and pseudo-second-order model rate constant, respectively.

The fitting curves are shown in Figure 4b,c, with the parameter shown in Table 2. The results show that the correlation coefficient (R^2^) of Hg(II) ion removal and the calculated value of *q_e_* via HA/Fe_3_O_4_/OS were 0.951 and 86.52 mg·g^−1^ in the pseudo-first-order model, while they were 0.998 and 90.45 mg·g^−1^ in the pseudo-second-order model, indicating that the experimental data fits the pseudo-second-order model better.

### 3.5. Adsorption Mechanism

**Figure 5:**
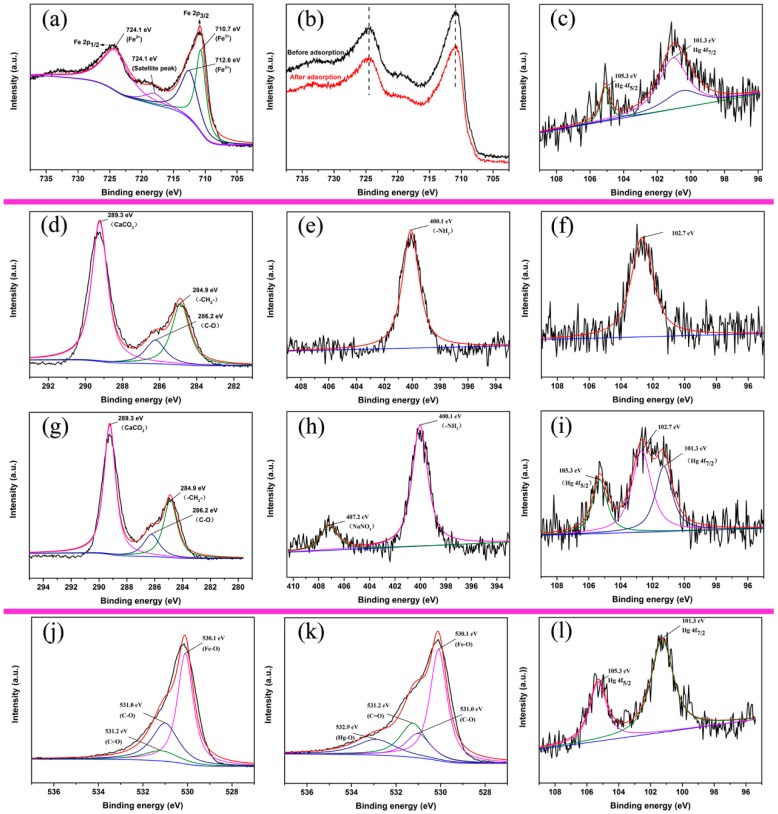
X-ray photoelectron spectroscopy (XPS) spectra of bare Fe_3_O_4_: Fe 2p (**a**) before and (**b**) after Hg(II) adsorption; (**c**) Hg 4f after adsorption. XPS spectra of pristine OS spectra: C 1s, N 1s, Hg 4f before ((**d**–**f**), respectively) and after ((**g**–**i**), respectively) Hg(II) adsorption. XPS spectra of OS/Fe_3_O_4_/HA: O 1s (**j**) before and (**k**) after Hg(II) adsorption; (**l**) Hg 4f after adsorption.

Fe_3_O_4_ is able to reduce some heavy metals due to the reducing capacity of Fe^2+^ [38], but whether the redox reaction is included in the removal of the mercury solution has not been determined. Also, OS has a complicated adsorption mechanism because of its special physical structure and complex organic composition [22]. Early literature reported that HA removed Hg(II) ions by a complexation reaction between carboxyl groups and mercury ions [39]. Due to the diversity of adsorption processes, XPS analysis was performed on the adsorbent before and after heavy metal ion removal to further explore the adsorption mechanism. The high-resolution XPS spectrum of Fe 2p from the synthetic material (Figure 5a) further proved the formation of Fe_3_O_4_ nanoparticles. The binding energy at Fe 2p_1/2_ had no insignificant change after Hg(II) ion removal (Figure 5b), indicating that the material had not been oxidized. Hg 4f spectra of Hg-loaded Fe_3_O_4_ nanoparticles could be fitted well with two doublet-peaks, namely 101.1 eV and 105.1 eV (Figure 5c). The distance between the 4f_7/2_ and 4f_5/2_ peaks (ΔeV) was 4.0 eV, which shows that the mercury adsorbed in Fe_3_O_4_ nanoparticles was considered as Hg(II) [40]. That is, the adsorption process of Fe_3_O_4_ nanoparticles was considered physical adsorption. C 1s spectra and N 1s spectra of pristine oyster shell before (Figure 5d,g) and after the reaction (Figure 5e,h) are shown. The C 1s spectra could be fitted into three peaks. The peaks of 284.9 eV and 286.2 eV may represent the functional group of –CH_2_– and C–O in organic composition, and the peak of 289.3 eV may represent the ingredient of CaCO_3_ [41]. There was a fitted peak at the binding energy of 400.1 eV, which was assigned to nitrogen of –NH_2_ [42]. Except for the addition of NO_3_– via mercury(II) nitrate standard solution, the high-resolution spectra of N 1s and C 1s were the same before and after the reaction. In addition, the appearance of 101.3 eV and 105.3 eV in Hg 4f spectra (Figure 5i) indicates that the mercury adsorbed in the oyster shell was present as Hg(II). The peak at 102.7 eV (Fig. 5f) may be the result of SiO_2_ in OS [43,44]. Thus, we can conclude that the function of OS in Hg(II) adsorption is to provide a porous structure to load the magnetic Fe_3_O_4_ nanoparticles, and the organic matter of OS plays a minor role in the adsorption. The high-resolution O 1s spectrum of OS/Fe_3_O_4_/HA reveals three peaks at binding energies of 530.1, 531.0, and 531.2 eV, as shown in Figure 5j, which were attributed to Fe–O, C–O, and C=O, respectively [45]. However, Figure 5k exhibits nonnegligible changes after loading mercury. The appearance of the peak at 532.9 eV indicates that the carboxyl group may have complexed with Hg(II) ions. The peaks of 101.3 eV and 105.3 eV in the Hg 4f spectrum (Figure 5l) further demonstrate the existence of chelation between the oxygen atom and Hg atom. In addition, the disappearance of the peak at 102.7 eV of OS may be due to the substantial wrapping by magnetic beads. The XPS analysis shows that the removal process of Hg(II) ions by OS/Fe_3_O_4_/HA involves physical adsorption and chemical adsorption.

### 3.6. Efficient and Selective Adsorption

**Figure 6:**
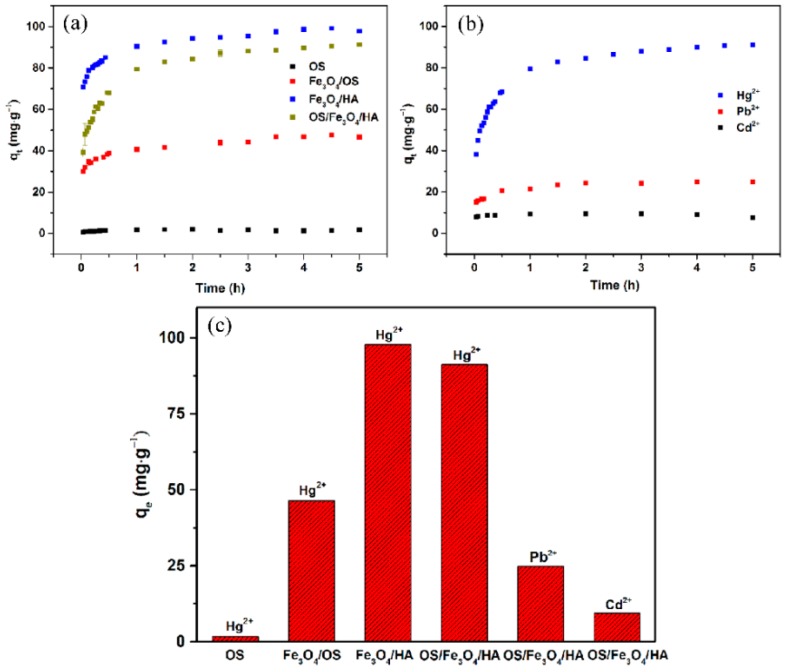
(**a**) The adsorption capacity of control groups and (**b**) of HA/Fe_3_O_4_/OS in various heavy metal solutions, and (**c**) a summarization (initial metal ion concentration, 25 mg·L^−1^, pH = 5).

The adsorption capacities of control groups were measured in the experiment (Figure 6a). The results show that the adsorption capacities of OS, Fe_3_O_4_/OS, and Fe_3_O_4_/HA were 1.8 mg·g^−1^, 46.5 mg·g^−1^, and 97.8 mg·g^−1^, respectively, while the adsorption capacity of Fe_3_O_4_ in previous literature was close to 20 mg·g^−1^ [46]. Furthermore, the preparation of OS/Fe_3_O_4_/HA was based on Fe_3_O_4_/HA—the only difference between them was the HA quantity, which was 0.5 g in Fe_3_O_4_/HA but 0.25 g in OS/Fe_3_O_4_/HA, with the replacement of 0.25 g of OS. However, the maximum adsorption capacity of OS/Fe_3_O_4_/HA was 91.2 mg·g^−1^, close to that of Fe_3_O_4_/HA. Previous literature studied the adsorption capacity of HA/Fe_3_O_4_ nanoparticle composites under different quantities of humic acid. It was revealed that HA could improve the adsorption capacity sharply [25]. Moreover, the nano-iron/oyster shell (NI/OS) composites were found to have better removal performance than NI in As^3+^ adsorption [24], which further proved the important role of OS as a carrier. To further discuss the adsorption performance, OS/Fe_3_O_4_/HA was compared to several different types of adsorbents (Table 3) [32,46,47,48,49,50]. Specifically, the maximal adsorption quantity of the thiol-modified Fe_3_O_4_@SiO_2_ was 148.8 mg·g^−1^, but the reaction rate was slightly slower than that of OS/Fe_3_O_4_/HA [46]. Furthermore, the maximal adsorption quantity of the glutamic acid-modified cellulose fibrous composite was only 22.9 mg·g^−1^, but it reached equilibrium very soon (within 10 min) [32]. OS/Fe_3_O_4_/HA, similar to the Lewatit MP 62, balancing the adsorption capacity and reaction rate to make the reaction occur quickly and efficiently [48]. In general, OS/Fe_3_O_4_/HA is a kind of adsorbent with an eminent adsorption capacity and a fast adsorption speed. Meanwhile, the utilization of OS helps in solving the problem of environmental pollution.

Non-selective adsorption has difficulties in removing specific heavy metal ions in complex industrial wastewater. With multiple heavy metal ions, the effective recycling of the target heavy metal ion is still a challenge. From the perspective of environmental protection and resource recovery, the preparation of an efficient adsorbent with specific adsorption really makes sense. In early 1980, Kerndorff added HA to a mixed solution that contained 11 metal ions (Hg^2+^, Fe^3+^, Pb^2+^, Cu^2+^, AI^3+^, Ni^2+^, Cr^3+^, Zn^2+^, Cd^2+^, Co^2+^, Mn^2+^) [51]. The study revealed that Hg(II) appeared to be integrated most firmly with HA.

In this study, the adsorption processes of Cd(II) ions and Pb(II) ions were also studied. Figure 6b shows the maximum adsorption capacities for Cd(II) ions and Pb(II) ions of 9.5 mg·g^−1^ and 24.9 mg·g^−1^, respectively, which were far less than the best adsorption capacity for Hg(II) ions. The large differences in adsorption capacity between different ions may be due to their different complex abilities. It was thus proven that OS/Fe_3_O_4_/HA can adsorb Hg(II) ions efficiently and specifically.

## 4. Conclusions

OS/Fe_3_O_4_/HA micro-nano material was successfully fabricated via the co-precipitation method. Due to its large specific surface area, material stability, and superparamagnetic properties, it exhibited superior removal capacity of mercury (91.2 mg·g^−1^). Meanwhile, it showed excellent selectivity and the maximum adsorption capacities for Cd(II) ions and Pb(II) ions were only 9.5 mg·g^−1^ and 24.9 mg·g^−1^, respectively. The XPS survey results show that the adsorption is a physicochemical adsorption. More specifically, OS acts as a carrier to load the fabricated nanoparticles, while both OS and the nanoparticles can physically adsorb mercury ions. Meanwhile, complexation exists between Hg(II) ions and the carboxyl group of HA. This study explored a novel adsorbent to remove Hg(II) ions. Moreover, it proposed an environmentally friendly route for the potential application of functionalized magnetic nanoparticles in environmental remediation.

## Figures and Tables

**Table 1:** Langmuir and Freundlich constants for the adsorption of Hg^2+^ ions via HA/Fe_3_O_4_/OS.

Langmuir Model			Freundlich Model		
*b* (L·mg^−1^)	*q_max_* (mg·g^−1^)	R^2^	*k_f_* (L·mg^−1^)	*n*	R^2^
0.18	141.57	0.991	19.81	1.55	0.968

**Table 2:** Adsorption kinetic parameters for the adsorption of Hg^2+^ ions via HA/Fe_3_O_4_/OS.

Pseudo-First-Order Model			Pseudo-Second-Order Model		
*k*_1_ (min^−1^)	*q_e_* (mg·g^−1^)	R^2^	*k*_2_ (g·mg^−1^·min^−1^)	*q_e_* (mg·g^−1^)	R^2^
0.1225	86.52	0.951	1.98 × 10^−3^	90.45	0.998

**Table 3:** Comparison of some adsorbents reported for adsorbing Hg^2+.^

Adsorbent	Equilibration Time (h)	*q_max_* (mg·g^−1^)	Reference
Thiol-functionalized magnetic activated carbon	~4 h	366.3	[47]
Lewatit MP 62	2 h	151.5	[48]
Purolite S 920	2 h	29.2	[48]
Thiol-modified Fe_3_O_4_@SiO_2_	4 h	148.8	[46]
Glutamic acid-modified cellulose fibrous	10 min	44.9	[32]
Carbon nanotube	~90 min	100	[49]
Coal-based activated carbon	~3 h	48.0	[50]
HA/Fe_3_O_4_/OS	~1 h	141.6	This work

## Supplementary Materials

The following are available online at https://www.mdpi.com/2079-4991/9/7/953/s1, Figure S1: Hysteresis loop of OS/Fe3O4/HA.
