# Supplementary material for: Preparation of Micro-Nano Material Composed of Oyster Shell/Fe3O4 Nanoparticles/Humic Acid and Its Application in Selective Removal of Hg(II)
Source: Nanomaterials (Basel). 2019 Jun 30;9(7):953. doi: 10.3390/nano9070953 (PMC6669493; doi:10.3390/nano9070953)
Supplement: Supplementary file 1 [file nanomaterials-09-00953-s001.pdf]

Supporting information

Preparation of Micro-Nano Material Composed of Oyster Shell/Fe_3O_4 Nanoparticles/Humic Acid and Its Application in Selective Removal of Hg(II)

Chuxian He [1,2], Junhao Qu [3], Zihua Yu [3], Daihuan Chen [3], Tiantian Su [3], Lei He [1], Zike Zhao [1], Chunxia Zhou [2,3,4], Pengzhi Hong [2,3,4], Yong Li [1], Shengli Sun [1] and Chengyong Li [1,2,4,*]

[1] School of Chemistry and Environment, Guangdong Ocean University, Zhanjiang 524088, China; chuxianhe0208@163.com (C.H.); airyhelei@163.com (L.H.); zzk64306460@163.com (Z.Z.); yongli6808@126.com (Y.L.); xinglsun@126.com (S.S.)

[2] Shenzhen Institute of Guangdong Ocean University, Shenzhen 518108, China; chunxia.zhou@163.com (C.Z.); hongpengzhi@126.com (P.H.);

[3] College of Food Science and Technology, Guangdong Ocean University, Zhanjiang 524088, China; 15975997422@163.com (J.Q.); m13005626792@163.com (Z.Y.); 13413673241@163.com (D.C.); sutiantiangdou@126.com (T.S.);

[4] Guangdong Modern Agricultural Science and Technology Innovation Center, Zhanjiang 524088, China

* Correspondence: cyli_ocean@163.com or cyli@gdou.edu.cn; Tel.: +86-759-239-6026

Materials

All chemicals were of analytical reagent grade used directly without any further purification. Iron (III) chloride hexahydrate ($FeCl_3 \cdot 6H_2O$) was obtained from Tianjin Fuchen chemical reagent factory, humic acid sodium salt was purchased from Aladdin Industrial Corporation (Shanghai). Nitric acid (65%, G.R.), Potassium hydroxide and Iron (II) sulfate heptahydrate ($FeSO_4 \cdot 7H_2O$) were supplied by Guangzhou chemical reagent factory, Potassium borohydride was purchased from Tianjin Kemiou Chemical Reagent Co., Ltd. Sodium hydroxide and Ammonium hydroxide (25%) were supplied by Guangdong Guanghua Sci-Tech Co., Ltd. Hg (1000 μg/ml, 5% HNO_3) was prepared by NCS Testing Technology Co., Ltd. Ultrapure water was used to prepare aqueous solution.

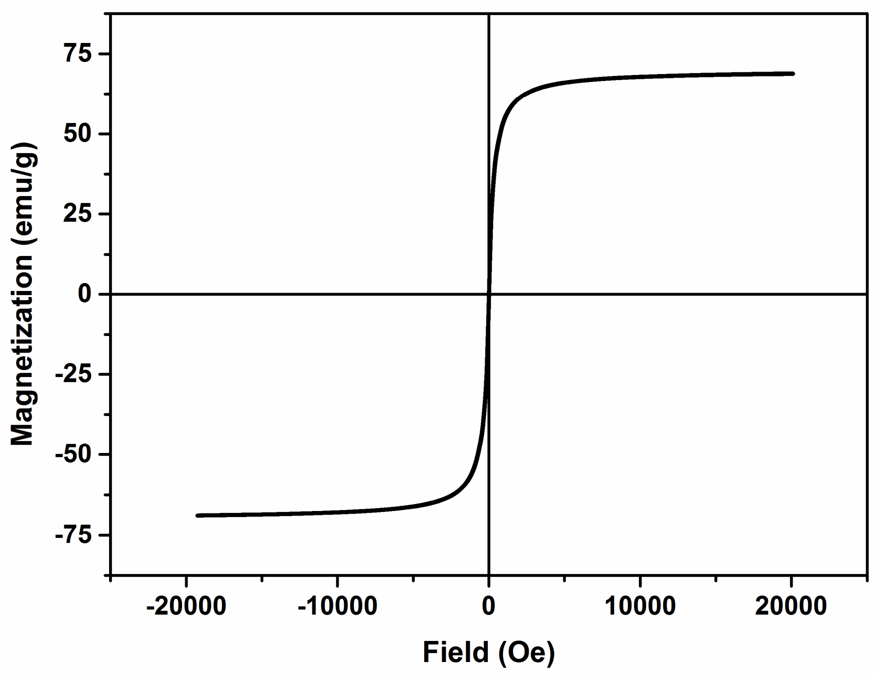

Figure S1. Hysteresis loop of OS/Fe_3O_4/HA.
